# The Anch'Or Harpoon Technique With a Manually Expandable Stentretriever (Tigertriever 13), a Technical Note

**DOI:** 10.3389/fneur.2022.934690

**Published:** 2022-07-26

**Authors:** Maud Wang, Stephanie Elens, Thomas Bonnet, Marin Halut, Juan Vazquez Suarez, Benjamin Mine, Boris Lubicz, Adrien Guenego

**Affiliations:** Department of Interventional Neuroradiology, Erasme University Hospital, Brussels, Belgium

**Keywords:** distal thrombectomy, stroke, anchor technique, Harpoon technique, endovascular recanalization

## Abstract

**Background and purpose:**

Stent and balloon anchor techniques have been described to obtain distal support and straighten catheter loops, stabilize microcatheters in giant aneurysms, or access distal tortuous anatomy during thrombectomy. These techniques require catheterization of distal arteries with a microcatheter but tortuosity and length issues may render it challenging, precluding the distal unsheathing of a classical auto-expandable stentretriever with the anchor technique.

**Methods:**

Therefore, we developed the so-called Anch'Or Harpoon Technique using a manually expandable stent retriever, the Tigertriever 13 (Rapid Medical, Yoqneam, Israel). Here, the stent retriever is not unsheathed but pushed out of a microcatheter, and then advanced as far as possible before manual opening.

**Results and conclusion:**

This technique may be used in 2 different situations. First, in the case of vessel tortuosity if the microcatheter can't be advanced as far as the physician wants: the Tigertriever 13 could be delivered through the microcatheter without having to unsheathe it, and be advanced and opened distally to its microcatheter to establish a stable anchor prior to advancing the guiding, intermediate, and micro-catheters (Anchor technique). The second situation is when distal occlusions lead to length issues; the microcatheter may be too short to cross a distal clot: the Tigertriever 13 could then be pushed out of the microcatheter, and be used to cross a sub-occlusive clot as it has a soft shaped distal tip and the physician has a visual on the artery beyond the sub-occlusion. Then, the Tigertriever would be manually expanded through the clot and retrieved (Harpoon technique) to obtain a recanalization.

## Introduction

Endovascular therapy (EVT) is an established treatment for acute ischemic stroke (AIS) patients with an anterior circulation large vessel occlusion (LVO) (1) and is now standard of care for such patients (2).

As a consequence, the number of cases grew exponentially within a few years, including patients with unusual and challenging anatomy, such as aortic and cervical loops, increasing the need for longer catheters; or patients with distal occlusions up to the fourth segment of the middle cerebral artery requiring specific low-profile microcatheters and stent retrievers (3, 4).

Different solutions exist to manage these situations such as different guiding and intermediate catheter lengths, radial artery instead of femoral access, or direct carotid puncture. Yet, carotid puncture appears safe only in intubated patients and requires a broad experience (5) with potential serious complications. Radial access may lead to similar efficacy (6) than femoral access and help avoiding aortic loops but it will not resolve the issue in case of cervical artery loops.

Despite carotid or radial access, one may still face issues in advancing catheters in case of challenging anatomy because of lack of distal support, or face a situation where despite classical anatomy and maximal advancement of both guiding and intermediate catheters, it would be impossible to cross a distal occlusion with a low-profile microcatheter to unsheathe a classical auto-expandable stent retriever because of length issues.

Therefore, we developed a novel strategy to get access to distal intracranial arteries in case of length issues during thrombectomy, or to get access to the extracranial and intracranial vasculature with the intermediate catheter in case of challenging anatomy, by advancing a manually expandable stent retriever, the Tigertriever 13 (Rapid Medical, Yoqneam, Israel) (7, 8), out of the microcatheter using its very low-profile and soft distal tip to safely catheterize previously inaccessible arteries and obtain distal support.

### Technical Note

We received approval from the local ethical standards committee (Ethics Committee Erasme Hospital, protocol P2021/062); the informed consent from participants was waived by Ethics Committee Erasme Hospital as all data were anonymized. The data that support the findings of this study are available from the corresponding author upon reasonable request. All methods were carried out in accordance with relevant guidelines and regulations.

### The Anchor Technique

A patient was referred to our academic hospital for a wake-up stroke, arriving at 2:35pm with a NIHSS of 22, a left hemiplegia, dysarthria, and hemineglect. A head computed tomography (CT), CT angiogram, and CT perfusion were performed at 2:45 pm showing a right proximal middle cerebral artery (M1) occlusion with an ischemic lesion of 52 ml (CBF < 30%), an Alberta Stroke Program Early CT Score (ASPECTS) of 2, a large mismatch of 85 ml with 137 ml of TMax > 6.0 s, and a mismatch ratio of 2.6 according to an automated software (RAPID, iSchemaView, Menlo Park). The patient was not eligible for IntraVenous injection of tissue Plasminogen Activator (IVtPA). Following recently published criteria (9), we opted for EVT after a multidisciplinary discussion and family information (core lesion <70 ml, mismatch ratio > 1.8, and mismatch volume > 15 ml) (9).

**Figure 1:**
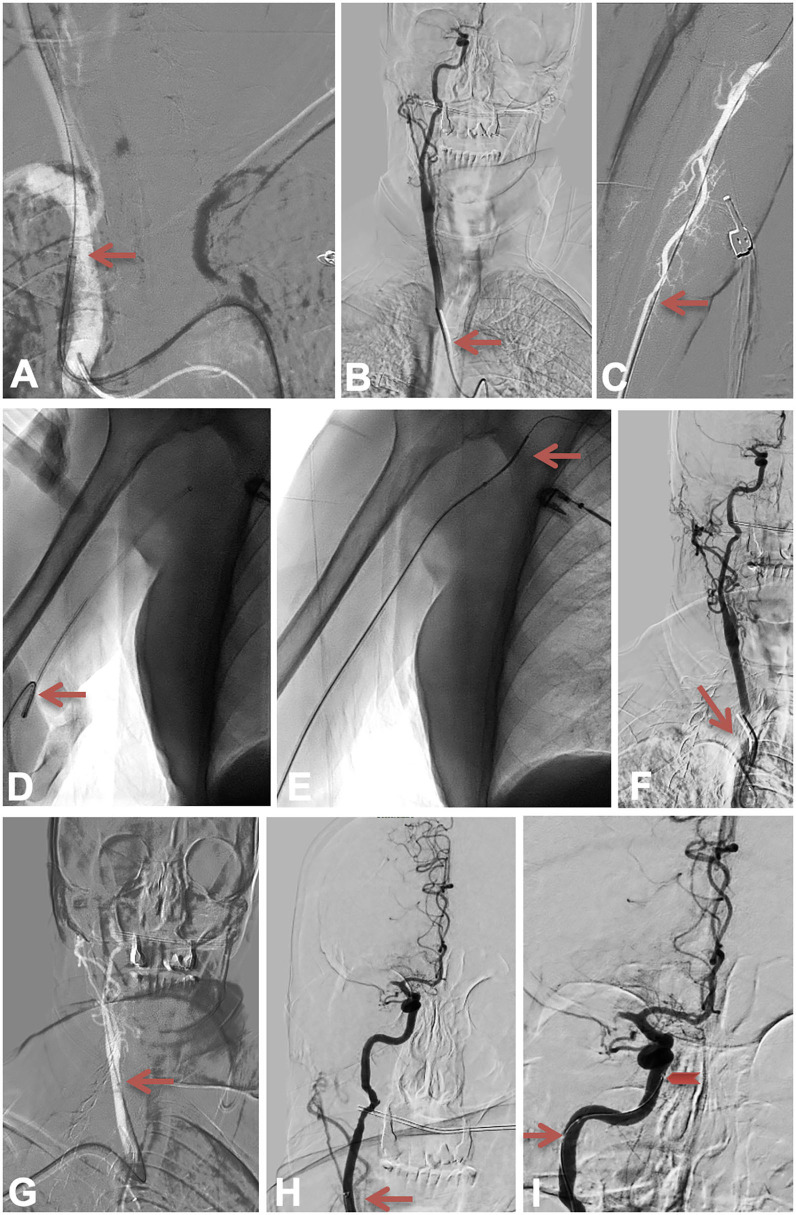
The anchor technique 1. Figure depicting the catheterization attempt through femoral access, then through right radial access, then the advancement of the guiding catheter, the intermediate catheter and the microcatheter in the right internal carotid artery. The red arrow in **(A)** shows the best position we managed to obtain with the main catheter through the femoral artery. The red arrow in **(B)** shows the SIM2 position. The red arrow in **(C–E)** is highlighting the radial access, and straightening of the humeral loop. The red arrow in (**F)** shows the SIM2 position while it shows the main catheter position in **(G)** and the intermediate catheter position in **(H)**. The red arrow in **(I)** shows how we managed to advance the microcatheter into the C5 intra-cavernous portion of the right ICA.

The patient entered the angioroom at 3:55 pm, the procedure was performed under local anesthesia. The right femoral artery was punctured at 4:18 pm under ultrasound, a short 9F femoral sheath was introduced. An 80 cm NEURONMAX (Penumbra, USA) was placed in the aorta over a 4F vertebral catheter and a 0.035 inch wire reaching the brachiocephalic trunk. The brachiocephalic trunk was first catheterized at 4:20 pm (see Figure 1A) but several attempts to catheterize the right common carotid artery failed because of tortuosity and a sharp angle between the aorta and the brachiocephalic trunk, suggesting a Type III arch. Several attempts were made using a 4F JB2, a 125 cm 5F JB2, a 125 cm 5F SIM 2 (Cordis, Cardinal Health) and several 0.035 and 0.035 Stiff wires, all attempts remained unsuccessful (see Figure 1B).

We decided not to puncture the right carotid artery because of the impossibility to achieve this specific arterial access without general anesthesia.

The right radial artery was punctured at 5:30 pm (see Figure 1C). A 90 cm 0.88 BALLAST (Balt, USA) was advanced, a right humeral loop was straightened using a 5F 125 cm SIM2 over a 0.035 Stiff wire (see Figures 1D,E), the right carotid artery was catheterized thanks to the SIM2 and a 0.035 wire (see Figure 1F). The BALLAST was advanced in the middle part of the right common carotid artery (see Figure 1G) but it was not possible to advance it further. A Sofia 6F (Microvention, Terumo, USA) was advanced in the cervical part of the right internal carotid artery (see Figure 1H) but attempts to advance it further failed despite several manipulations. A Headway 27 (Microvention, Terumo, USA) microcatheter was then advanced over a Traxcess 14 (Microvention, Terumo, USA) microwire, then a Transend 14 microwire (Stryker, Kalamazoo, MI, USA). We managed to advance it into the C5 intra-cavernous portion of the right ICA but not further (see Figure 1I), and despite managing to get the J-shaped microwire up to the right M2, we were not able to advance any catheter further over it.

**Figure 2:**
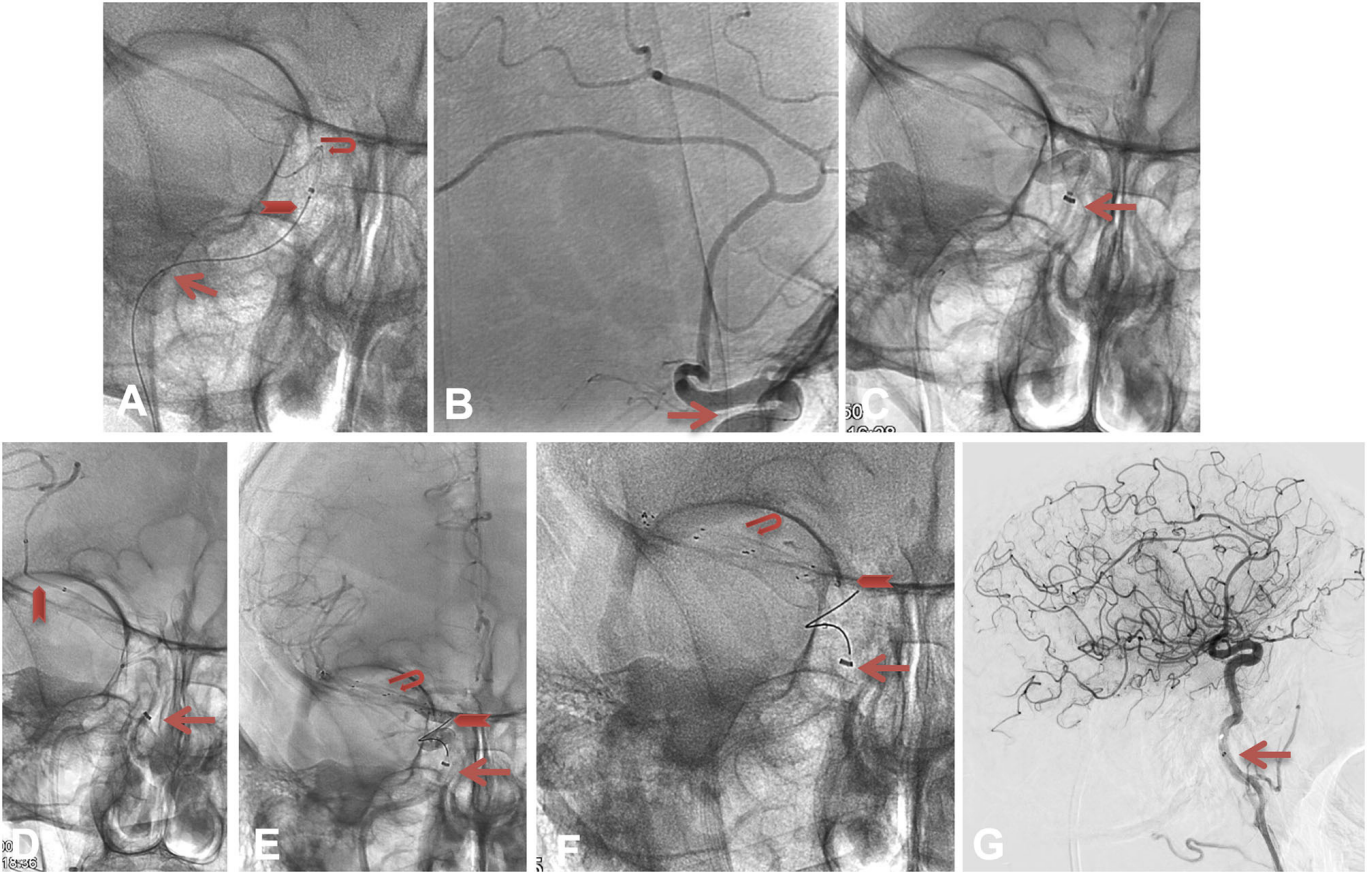
The Anchor technique 2. The red arrow in **(A,B)** shows the position of the microcatheter's markers, and the closed Tigertriever 13 in the carotid siphon. To avoid any risk on the M1 perforators or anterior choroidal artery, we opened the Tigertriever 13 in the C3–C4 segment [position seen in **(B)**] and then, managed to advance the microcatheter and the intermediate catheter in the cavernous ICA **(C)**. We advanced the microcatheter over a J-shaped microwire beyond the occlusion, deployed a 6 × 40 mm Solitaire (Medtronic, Minneapolis, USA) and obtained a mTICI 2b recanalization at 6:22 pm [see **(D–G)**].

We decided to use a low-profile manually expandable stent retriever, the Tigertriever 13, as an anchor in the cavernous ICA (see Figures 2A,B). To avoid any risk on the M1 perforators or anterior choroidal artery, we opened the Tigertriever 13 in the C3–C4 segment and then managed to advance the microcatheter and the intermediate catheter in the cavernous ICA (see Figure 2C).

We advanced the microcatheter over a J-shaped microwire beyond the occlusion, deployed a 6 × 40 mm Solitaire (Medtronic, Minneapolis, USA) and obtained an mTICI 2b recanalization at 6:22 pm (see Figures 2D–G).

The patient had an immediate post-EVT and 24 h post-EVT head CT showing a stable ischemic lesion, without hemorrhagic transformation. The NIHSS improved to 15 on day 1, the patient was discharged to rehabilitation with a mRS of 4 at day 6, we currently do not have any further follow up.

### The Harpoon Technique

A patient was referred at our academic hospital from a regional stroke center after having developed a right hemiplegia with aphasia at 1 pm, with an NIHSS of 15. The head CT, CT angiogram, and CT perfusion were performed outside our facility at 2:30 pm, showing a left distal ICA occlusion with no ischemic lesion (CBF < 30%), an Alberta Stroke Program Early CT Score (ASPECTS) of 10, a large mismatch and TMax > 6.0 s of 234 ml, and an “infinite” mismatch ratio according to an automated software (RAPID, iSchemaView, Menlo Park). The IVtPA was not performed because of an active anticoagulant therapy. The patient was transferred and arrived at our academic hospital angioroom at 4:40 pm, with an NIHSS of 12.

**Figure 3:**
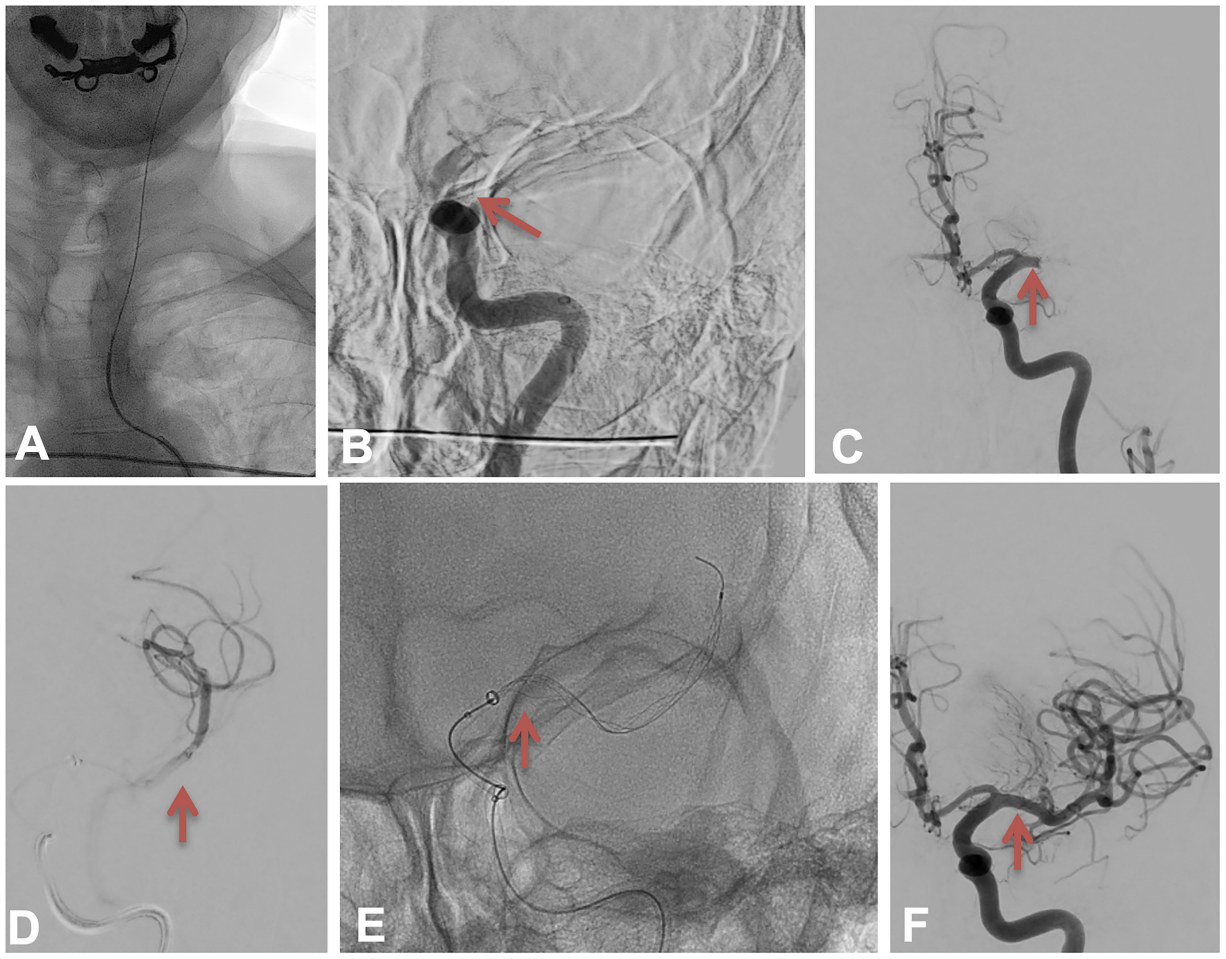
The Harpoon technique 1. **(A)** depicting the left internal carotid artery catheterization. The red arrow in **(B)** depicts the left internal carotid artery occlusion at the beginning of the procedure. The red arrow in **(C)** shows the M1 occlusion after a first pass. The red arrow in **(D)** depicts the run realized over the microcatheter in M2 branches. The red arrow in **(E)** shows the position of the Tigertriever XL covering the M1 clot and how the intermediate catheter was advanced to pinch the stent retriever. The red arrow in **(F)** shows the recanalization after a second pass.

The procedure started under local anesthesia. The right femoral artery was punctured at 4:45 pm under ultrasound, a short 9F femoral sheath was introduced. An 80 cm NEURONMAX (Penumbra, USA) was placed in the aorta over a 5F JB2 catheter with a 0.035-inch wire, the left common carotid artery was catheterized (see Figure 3A). A Sofia 6F (MicroVention, Terumo, USA) was advanced in the cervical ICA, an angiogram showed the distal ICA occlusion (see Figures 3B,C). The intermediate catheter was advanced over a Headway 27 microcatheter (MicroVention, Terumo, USA) and a J-shaped Transend 14 microwire (Stryker, Kalamazoo, MI, USA). We crossed the occlusion and deployed a Tigertriever XL (Rapid Medical, Yoqneam, Israel), performed a contact aspiration (no image shown) while retrieving the stent retriever and obtained an ICA recanalization at 4:58 pm with a persisting left M1 occlusion (see Figure 3C).

**Figure 4:**
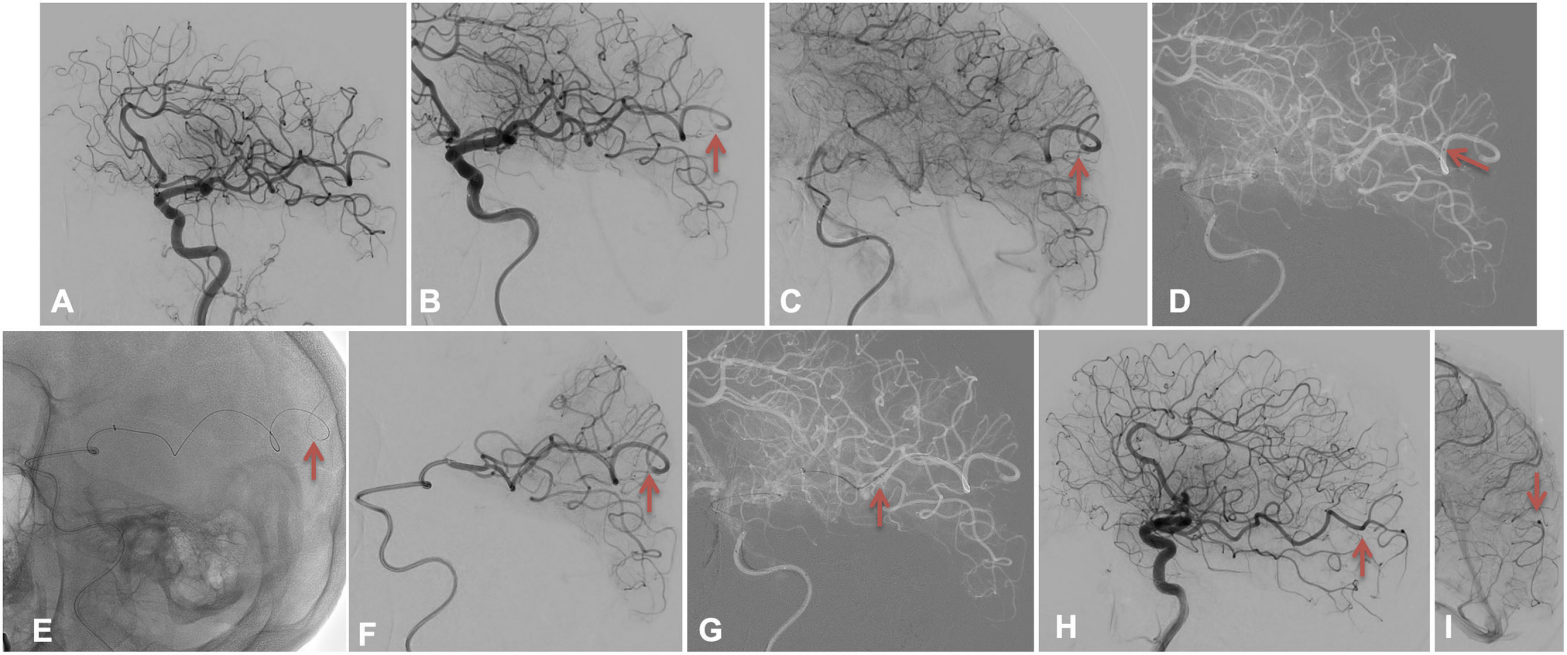
The Harpoon technique 2. **(A–C)** depicts the left M4 distal sub-occlusion using red arrows; the red arrow in **(D)** shows the most distal position of the microcatheter, while it shows the harpoon technique performed by advancing the Tigertriever 13 in the M4 artery beyond the microcatheter as it was too short in **(E,F)**. The red arrow in **(G)** shows the retrieval of the stentretriever and microcatheter. A complete recanalization TICI 3 is highlighted in **(H,I)** by the red arrows.

We decided to switch to general anesthesia because of the patient's agitation. The procedure started again at 5:33 pm. The intermediate catheter was advanced over a Headway 27 microcatheter (Microvention, Terumo, USA) and a J-shaped Transend 14 microwire (Stryker, Kalamazoo, MI, USA), we crossed the occlusion and deployed the Tigertriever XL (Rapid Medical, Yoqneam, Israel), performed a contact aspiration while retrieving the stent retriever (see Figures 3D,E) and obtained an M1 recanalization at 5:38 pm (see Figure 3F) with a persisting distal M4 occlusion (see Figures 4A–C).

After discussing the matter with our team, as the patient did not undergo IVtPA and was under general anesthesia, we decided to perform a distal EVT.

Using a Headway Duo microcatheter (MicroVention, Terumo, USA) over an Hybrid 0.008 microwire (Balt, USA), the left M4 branch was catheterized (see Figure 4D), and the intermediate catheter was advanced as far as possible in the MCA. Unfortunately, the microcatheter was too short and remained proximal to the M4 occlusion (see Figure 4D). A distal run was performed to confirm the persisting partial occlusion, and a severe arterial delay beyond the clot. A manually expandable low-profile stent retriever, the Tigertriever 13, was loaded and gently advanced through the clot as we had a visual on the exact position of the occlusion and the branches beyond the occlusion (Harpoon technique), without feeling any resistance (see Figure 4E and Supplementary Video 1). We opened it through the clot (see Figure 4F) and retrieved it (see Figure 4G and Supplementary Video 2) after 2 min. A complete recanalization TICI 3 (see Figures 4H,I) was obtained at 5:55 pm.

Day 1 NIHSS was 3, no hemorrhagic transformation was seen on day 1 head CT, patient was discharged at day 4 with a NIHSS of 1, mRS 2. The 3 months mRS was 1.

**Figure 5:**
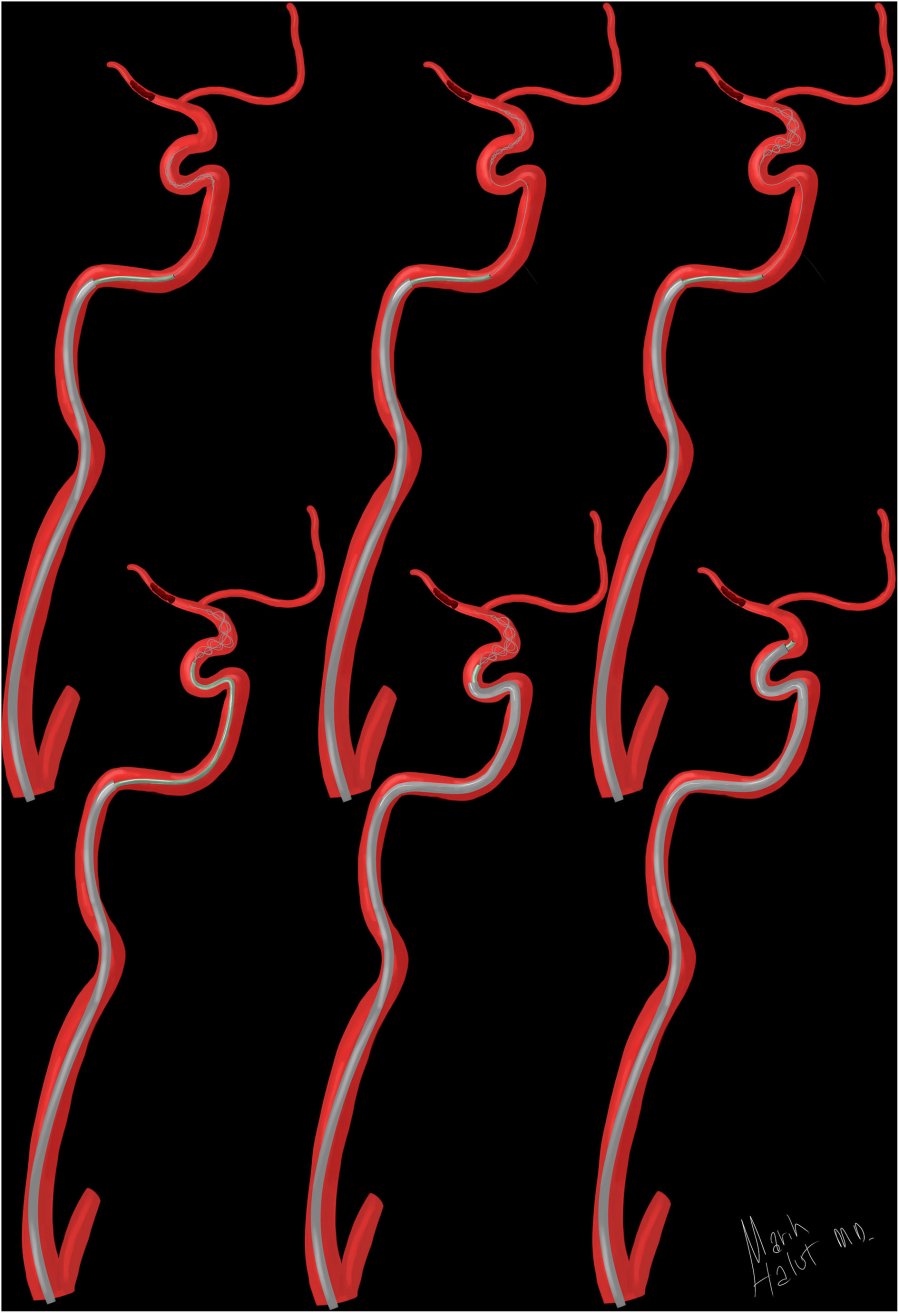
Illustration of the Anchor technique. Figure depicting how the Tigertriever 13 can be advanced out of the microcatheter in the Anchor technique, opened in a safe arterial segment to gain support and used to advance both the microcatheter and intermediate catheter.

**Figure 6:**
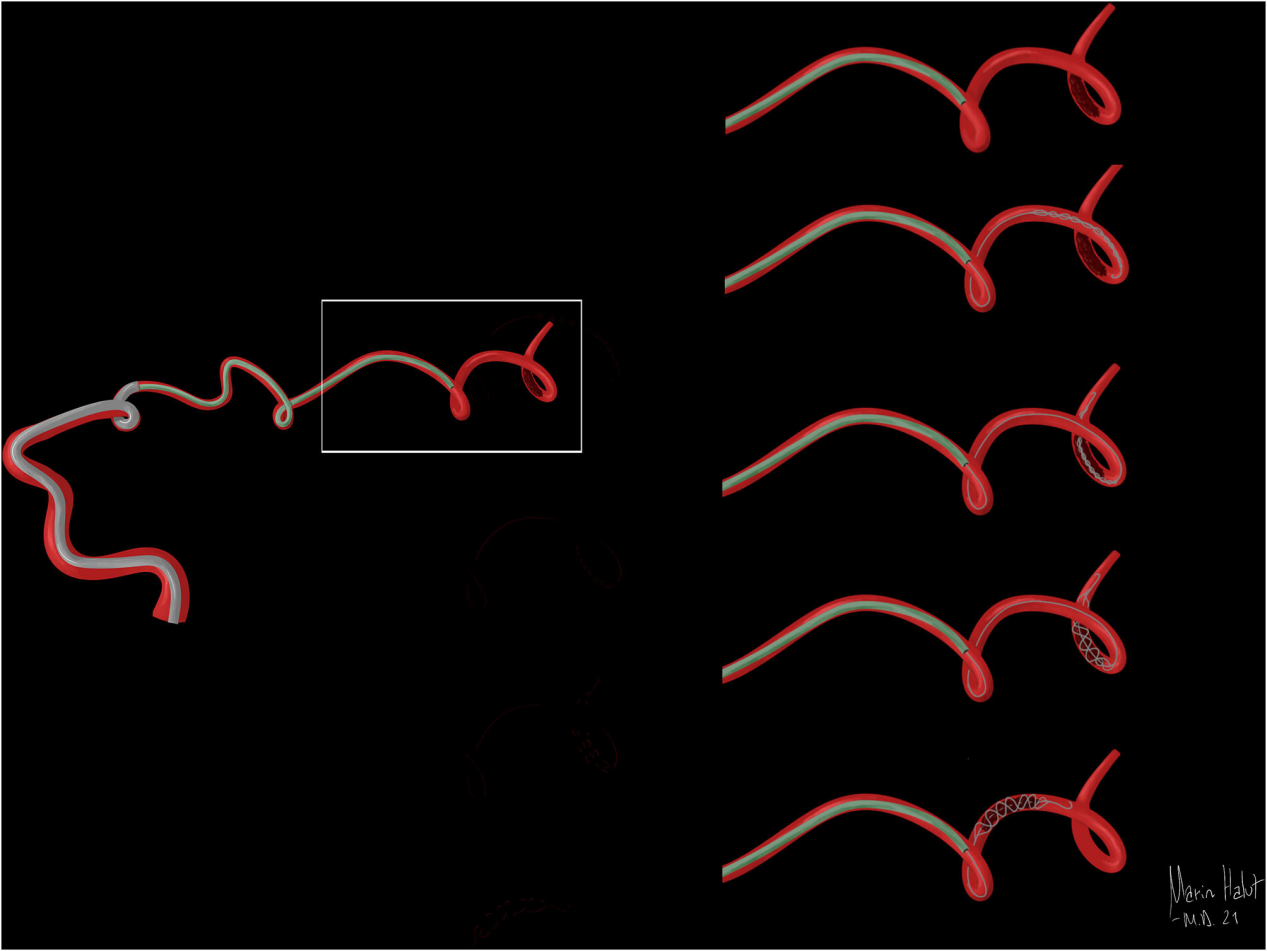
Illustration of the Harpoon technique. Figure depicting how the Tigertriever 13 is used in the Harpoon technique to reach a distal clot when the microcatheter length does not let us unsheathe it through the occlusion.

Figures 5, 6 are illustrating how both techniques can be performed using the Tigertriever 13, as explained.

## Discussion

As a result of large randomized trials, more and more patients are treated for AIS with LVO, leading to an increase in challenging cases with difficult anatomy and long catheterization times.

The advantage of the anchor technique has already been illustrated for large aneurysms (10–14) and stroke thrombectomy (15, 16) with both auto-expandable stent retrievers (12, 15–17) and balloons (10, 14).

Yet, in all these cases, the neurointerventionist must be able to catheterize the targeted intracranial artery using a microcatheter or balloon, which can occasionally/sometimes be challenging.

The Tigertriever 13 is a stentriever comprised of a fine wire mesh mounted on a flexible shaft that expands when the physician pulls a control wire that is connected to its distal end (18, 19). The design of the wire mesh is optimized to penetrate the clot and encapsulate it during retrieval. The mesh is constructed from nitinol wires, the distal end of the device consists of a flexible tip, allowing a gentle and safe navigation (18, 19). Tigertriever 13's low profile enables delivery through a microcatheter with an internal diameter of 0.0165 inches. Its controllability allows for adaptable control of the device in order to minimize the resistance during clot retrieval.

Thanks to these properties, the Tigertriever 13 was used in our case applying the anchor technique and gently advanced in the intra-cavernous ICA to ensure no resistance was felt, under continuous fluoroscopy, then opened to obtain more support to advance the guiding and intermediate catheter. Other stent retrievers could have been used in this situation as described in other anchor technique manuscripts, but as most of them are self-expandable, the physician needs to unsheathe them, which means pulling back the microcatheter and taking the risk to lose the access in a difficult situation. We thought the manually expandable design of the Tigertriever would decrease this risk.

Recently, consensus statements have been proposed on the distal and medium occlusions as it is considered to be the new endovascular frontier (20). Several devices are being used with a good efficacy and safety (21–23) in such indications, the Tigertriever 13 being one of them. In our case, an 80 cm guiding catheter and 6F 131 cm intermediate were advanced as far as possible but the 6F was blocked in the M2 segment because of the artery diameter. The 156 cm Headway Duo was advanced as far as possible but did not reach the occlusion. As we were proximal to the sub-occluded M4 cortical branch and the branches beyond the sub-occlusion were visible, we decided to gently advance the Tigertriever 13 using its distal tip as a wire (Harpoon technique). We ensured that no resistance was felt under continuous fluoroscopy and that the distal tip did not block itself within the clot or further.

Other solutions would have been to pull back the intermediate catheter and use the microcatheter alone, use a shorter 5F intermediate catheter instead of a 131 cm 6F intermediate catheter, or use a 167 cm Headway Duo. Now we would typically use a 167 cm Headway Duo for such a situation as it is the softest and smallest diameter microcatheter that can be used with this device, and avoid bare navigation of the stent retriever. This extremely distal lesion was not appropriate for the Headway Duo 156 and bare navigation of a stent retriever in distal vessel segments should not be recommended.

The manually expandable design of the Tigertriever 13 offers new perspectives, which have to be carefully evaluated in terms of safety and success in the future. Different improvements of the device could be considered, for example, a longer, stiffer, shapeable distal tip; or a moveable wire independent of the stent retriever.

## Conclusions

The Anch'Or Harpoon Technique using the manually expandable stent retriever Tigertriever 13 in which the stent retriever is advanced beyond the microcatheter tip may be a potential alternative to previously described techniques, exchange maneuvers or carotid puncture in case of challenging anatomy or length issues; however, bare navigation of a stent retriever in distal vessel segments should not be recommended.

## Data Availability Statement

The raw data supporting the conclusions of this article will be made available by the authors, upon reasonable request.

## Ethics Statement

The studies involving human participants were reviewed and approved by Erasme Ethical Committee. Written informed consent for participation was not required for this study in accordance with the national legislation and the institutional requirements.

## Author Contributions

MW, SE, TB, MH, JS, BM, BL, and AG participated to study design, data collection, data analysis, and writing. All authors contributed to the article and approved the submitted version.

## Conflict of Interest

The authors declare that the research was conducted in the absence of any commercial or financial relationships that could be construed as a potential conflict of interest.

## Publisher's Note

All claims expressed in this article are solely those of the authors and do not necessarily represent those of their affiliated organizations, or those of the publisher, the editors and the reviewers. Any product that may be evaluated in this article, or claim that may be made by its manufacturer, is not guaranteed or endorsed by the publisher.

## Abbreviations

AIS: acute ischemic stroke
ASPECTS: Alberta Stroke Program Early CT Score
CT: computed tomography
EVT: endoVascular therapy
IVtPA: intravenous injection of tissue plasminogen activator
LVO: large vessel occlusion
mRS: modified Rankin Scale
NIHSS: National Institute of Health Stroke Scale
mTICI: modified thrombolysis in cerebral infarction.
